# Developing a framework to guide the de-adoption of low-value clinical practices in acute care medicine: a study protocol

**DOI:** 10.1186/s12913-017-2005-x

**Published:** 2017-01-19

**Authors:** Jeanna Parsons Leigh, Daniel J. Niven, Jamie M. Boyd, Henry T. Stelfox

**Affiliations:** 1 Department of Critical Care Medicine, University of Calgary, Calgary, AB Canada; 2 Alberta Health Services, Alberta, Canada; 3 O’Brien Institute for Public Health, University of Calgary, Calgary, AB Canada; 4 Department of Community Health Sciences, University of Calgary, Calgary, AB Canada; 5 Department of Medicine, University of Calgary, Calgary, AB Canada

**Keywords:** De-adoption, Low-value practices, Barriers, Facilitators, Acute care medicine

## Abstract

**Background:**

Healthcare systems have difficulty incorporating scientific evidence into clinical practice, especially when science suggests that existing clinical practices are of low-value (e.g. ineffective or harmful to patients). While a number of lists outlining low-value practices in acute care medicine currently exist, less is known about how best to initiate and sustain the *removal* of low-value clinical practices (i.e. de-adoption). This study will develop a comprehensive list of barriers and facilitators to the de-adoption of low-value clinical practices in acute care facilities to inform the development of a framework to guide the de-adoption process.

**Methods:**

The proposed project is a multi-stage mixed methods study to develop a framework to guide the de-adoption of low-value clinical practices in acute care medicine that will be tested in a representative sample of acute care settings in Alberta, Canada. Specifically, we will: 1) conduct a systematic review of the de-adoption literature to identify published barriers and facilitators to the de-adoption of low-value clinical practices in acute care medicine and any associated interventions proposed (*Phase one*); 2) conduct focus groups with acute care stakeholders to identify important themes not published in the literature and obtain a comprehensive appreciation of stakeholder perspectives (*Phase two*); 3) extend the generalizability of focus group findings by conducting individual stakeholder surveys with a representative sample of acute care providers throughout the province to determine which barriers and facilitators identified in *Phases one* and *two* are most relevant in their clinical setting (*Phase three*). Identified barriers and facilitators will be catalogued and integrated with targeted interventions in a framework to guide the process of de-adoption in each of four targeted areas of acute care medicine (Emergency Medicine, Cardiovascular Health and Stroke, Surgery and Critical Care Medicine). Analyses will be descriptive using a combination of qualitative and quantitative analyses.

**Discussion:**

There is a growing body of literature suggesting that the de-adoption of ineffective or harmful practices from patient care is integral to the delivery of high quality care and healthcare sustainability. The framework developed in this study will map barriers and facilitators to de-adoption to the most appropriate interventions, allowing stakeholders to effectively initiate, execute and sustain this process in an evidence-based manner.

**Electronic supplementary material:**

The online version of this article (doi:10.1186/s12913-017-2005-x) contains supplementary material, which is available to authorized users.

## Background

The challenge of implementing best evidence into clinical practice is a significant problem in modern healthcare [1] that can result in inappropriate, ineffective, inefficient and unsafe care. This is particularly problematic in acute care facilities, where large numbers of expensive therapies, technologies and treatments are used daily in an effort to save lives [2, 3]. There is a growing body of literature [4] which suggests that the removal (i.e. **de-adoption**) of ineffective and potentially harmful (i.e. **low-value**) clinical practices is integral to the delivery of high quality care and the sustainability of our healthcare system [5–8]. This is highlighted by the growing number of initiatives—such as the Choosing Wisely Campaign®—designed to reduce the use of low-value practices. Previous studies have produced a conceptual model for de-adoption, [4] and list of candidate practices to be de-adopted [9]. However, few studies have examined how to successfully de-adopt an intervention that is already integrated into clinical practice [4]. Thus, while there is an urgent need to de-adopt practices that are of low-value, the process of de-adoption remains poorly understood.

### The challenge of translating research into clinical practice

Translating research evidence into clinical practice is important, especially for patients admitted to acute care facilities where expensive and rapidly evolving technologies are used to save lives and reduce suffering. Yet the lag between the development of scientific evidence through randomized controlled trials and the implementation of that evidence into routine clinical practice remains significant (~17 years) [10]. The Institute of Medicine has identified six contributing factors: **1)** limited knowledge of how to implement evidence into practice; **2)** inefficient methods of dissemination; **3)** limited evidence on comparative effectiveness to guide investments and use of technologies; **4)** absence of national standards of care; **5)** research groups and clinical communities working in isolation; and **6)** inadequate assessment of the impact of cost, societal values and personal preferences [11, 12]. This results in three types of knowledge-practice gaps:[11, 13] **over use** (prescription of an intervention despite scientific evidence indicating it is ineffective or harmful), **under use** (little to no prescription of an intervention that science has shown to be effective) and **misuse** (prescription of an intervention that scientific evidence has shown to be effective, but it is prescribed for the wrong patients, under the wrong circumstances), which have important implications for patient care and outcomes [14].

### The case for a focus on acute care facilities

Hospital-based care is a critical component of healthcare [15]. Hospitals provide necessary treatment to patients with life and limb threatening illnesses, and play a vital role in the prevention of morbidity and mortality [16]. Patients treated in acute care facilities are the sickest in the healthcare system, with a wide range of complex medical problems that require urgent treatment (e.g. casting fractures, complex surgical and medical interventions, life support technologies in intensive care units [ICU], etc.). In 2011, acute care facilities across member countries of the Organization for Economic Co-Operation and Development (OECD) recorded approximately 31 emergency department visits per 100 members of the population, marking a 5.2% increase in the total number of visits to emergency departments over the past decade [17]. At the same time, hospitalisation rates have increased in about one-third of OECD countries, with curative or rehabilitative inpatient care accounting for more than a third of health spending in some countries [18]. Rising utilization, resource constraints and resultant capacity strain threaten the current state of our acute care systems [19]. Moreover, translating research evidence into best practice is especially problematic in the care of acutely ill patients where treatment is time sensitive and multiple expensive tests, treatments and technologies are concomitantly used in an attempt to preserve life or limb [20]. Given existing fiscal climates, aging populations, and the proliferation of advanced clinical treatments, utilization of services may exceed the financial and human resource capacities of our healthcare system [21, 22].

### The need to identify and de-adopt low-value clinical practices

Knowledge translation (KT) is a continuous quality improvement process largely focused on *getting evidence into practice* (i.e. **adoption**) [23]. However, burgeoning emphasis has recently been placed on the advancement of healthcare agendas that preference the removal (i.e. **de-adoption**) of health technologies and clinical practices that are of low-value (i.e. ineffective or harmful to patients), given the strong potential to reduce preventable harm, simplify care, and reallocate resources to effective, high-value therapies. The push to expand KT activities to include de-adoption is essential, as challenges abound when it comes to the removal of established therapies no longer supported by evidence. For example, a number of studies published in top clinical journals [24, 25] have demonstrated that fluid resuscitation with albumin has no effect on meaningful clinical outcomes for the majority of patients, and is harmful when used in patients with traumatic brain injury [24]. However, many patients (including some with traumatic brain injury) continue to be prescribed albumin [26]. Given that a growing body of research suggests that low-value practices are common in medicine, [4] and that the existence and widespread publication of new, more robust evidence has repeatedly been shown to fall short of catalysing important clinical practice change, [27, 28] the challenge is to find effective ways to initiate, execute and sustain a process of de-adoption to improve quality and value in healthcare.

In direct response to this challenge, this study will catalogue top barriers and facilitators to de-adoption in the development of an applied framework to guide the systematic de-adoption of low-value clinical practices in acute care medicine. Specifically, the existing evidence base of published barriers and facilitators to de-adoption will be analyzed to inform stakeholder focus groups and the development of a survey instrument to determine top barriers and facilitators to de-adoption in a representative sample front line providers and decision-makers from 14 acute care facilities across Alberta, Canada. The outcome will be an applied strategy to facilitate the removal of practices that are no longer supported by best evidence.

## Objectives

### Overarching objective

To develop a strategy to guide the de-adoption of low-value clinical practices within adult acute care medicine. We intend to accomplish this objective through:

### Specific objectives

1. A systematic review of evidence-based barriers and facilitators to de-adoption and associated interventions.
2. An assessment of the top barriers and facilitators to de-adoption in a single healthcare system.
3. The development of a framework that integrates barriers and facilitators with specific interventions to guide the process of de-adoption.

## Methods/Design

### Study design

We propose to identify and catalogue the top barriers and facilitators to the de-adoption of low-value clinical practices in a representative sample of adult acute care settings in Alberta in an effort to build an evidence-based applied framework that will guide the process of de-adoption. This three phase mixed methods study is the initial step in a program of research that will launch and sustain a systematic and customizable de-adoption strategy in adult acute care medicine. The framework of barriers and facilitators resulting from this project will ultimately support clinical and health policy decision-making and improve the quality of care patients receive. In line with the World Health Organization (WHO), we define acute care as a clinical service that responds to immediately life- or limb-threatening health conditions, encompassing a range of clinical healthcare functions, including emergency care, urgent care, short-term stabilization, pre-hospital care, critical care and trauma care & acute care surgery [16]. Moreover, we divide low-value clinical practices into two categories: those that are ineffective (i.e. do not produce any significant or desired effect) and those that are harmful (i.e. where the risk of harm outweighs any benefit).

To ensure feasibility of the study we have restricted our focus to four areas of specialization in acute care medicine—Emergency, Cardiovascular Health and Stroke, Surgery and Critical Care. Our sampling frame will consist of the 14 adult acute care facilities in Alberta, Canada that have an Emergency Department, Operating Room, Cardiovascular Coronary Care Unit and Intensive Care Unit. These hospitals were selected because they will provide a population-based sample representative of the broad spectrum of tertiary, teaching, and community settings in the Alberta acute care landscape. Moreover, Alberta is an ideal location to pilot test this work because the recent overhaul of health system governance in the province for quality and safety has led to the establishment of a province-wide tapestry of integrated Strategic Clinical Networks (SCNs) [29] whose implementation has created a mandate and mechanism to conduct applied population-based research. The SCNs are provincial teams of healthcare providers, researchers, government representatives and patients and families tasked with developing a strategy to strengthen clinical engagement and improve the delivery of evidence-informed care. We intend to use this unique environment as a ‘living laboratory’ to evaluate published barriers and facilitators to de-adoption. Furthermore, while this work will focus on adults, future work could be extended to pediatrics and neonatology.

**Table:** 

ᅟ	ᅟ
**Study sites for data collection** • Foothills Medical Centre, Calgary, Alberta • Peter Lougheed Centre, Calgary, Alberta • Rockyview General Hospital, Calgary, Alberta • South Health Campus, Calgary, Alberta • Red Deer Regional Hospital, Red Deer, Alberta • Grey Nuns Community Hospital, Edmonton, Alberta • Misericordia Community Hospital, Edmonton, Alberta • Royal Alexandra Hospital, Edmonton, Alberta • University of Alberta Hospital, Edmonton, Alberta • Sturgeon Community Hospital, St. Albert, Alberta • Northern Lights Regional Health Centre, Fort McMurray, Alberta • Queen Elizabeth II Hospital, Grand Prairie, Alberta • Chinook Regional Hospital, Lethbridge, Alberta • Medicine Hat Regional Hospital, Medicine Hat, Alberta	

### Approach

**Fig. 1:**
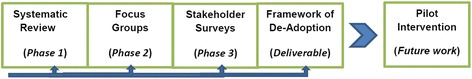
Methodological schema

The proposed study aims to develop a framework to guide de-adoption in acute care settings using a three phased approach: **1)** we will build on our team’s previously completed scoping review [4] to conduct a systematic review of the barriers and facilitators to de-adoption (*Phase one*); **2)** we will conduct focus groups with acute care stakeholders to identify important barriers and facilitators not published in the literature and obtain a comprehensive appreciation of stakeholder perspectives; (*Phase two*); **3)** we will extend the generalizability of focus group findings by conducting individual stakeholder surveys with a representative sample of acute care providers throughout the province to allow us to better understand barriers and facilitators to de-adoption (*Phase three*). Analyses will be descriptive using a combination of qualitative and quantitative analyses (Fig. 1).

### Systematic review (Phase one)

#### Objective

To conduct a systematic review of published barriers and facilitators to the de-adoption of low-value clinical practices in adult acute care medicine and associated interventions listed as effective. This protocol was developed according to the Preferred Reporting Items in Systematic Reviews and Meta-analyses (PRISMA) statement, [30] has been registered in PROSPERO International Prospective Register of Systematic Reviews (CRD42016050234) (Additional file 1). We define barriers and facilitators as factors that impede or promote use of best evidence in clinical practice. We define de-adoption as the discontinuation of a clinical practice after it has been previously adopted.^4^ A comparison group is not required as we will be looking for studies that describe barriers and facilitators including observational studies. However, if evaluation studies are identified, details on the comparison group will be assessed including patients and care setting.

#### Search strategy

The search strategy was designed using our existing published scoping review of de-adoption, [30] updating the systematic search and restricting our focus to citations describing barriers and facilitators. To update this review we will search the following electronic databases from March 5, 2014 to Fall 2016: PubMed, Medline, EMBASE, CINAHL and the Cochrane Library. A search of the ‘grey’ literature will also be conducted using the CADTH tool for searching grey literature. Bibliographies of retrieved articles will be searched for additional relevant articles. Additional information will be requested from study authors if necessary.

The search terms are specific to articles reporting de-adoption, including text words that include combinations and synonyms of *de-adoption* and *healthcare technologies*. Appropriate wildcards will be used to account for plurals and variations in spelling. Appendix outlines the proposed MEDLINE search strategy.

#### Eligibility criteria

We will include English language citations that refer to barriers or facilitators to the de-adoption of any clinical practice in adult patients (age ≥ 18 years) with medical, surgical, or psychiatric illnesses in any healthcare setting. All original and non-original quantitative and qualitative research citations will be eligible.

#### Outcomes

The exact list of outcomes is unknown; however, it is likely to include: barriers, facilitators and interventions designed to target barriers or complement facilitators. We define barriers and facilitators as factors that influence the discontinuation of a clinical practice after it has been previously adopted

#### Citation selection

Prior to the screening of titles and abstracts, the citation screening form will be calibrated through pilot testing. Two investigators will independently review a random sample of 50 citations from the literature search, and the inclusion/exclusion criteria will be serially revised until citation selection is reliable (k > = 0.8). The same two investigators will then independently review citations through a two-step process. First, the titles and abstracts of all citations will be screened against inclusion criteria. Second, the full text of any citation categorized as include or unclear by either investigator will be reviewed to determine whether it meets eligibility criteria. Eligibility disagreements will be resolved between the reviewers by consensus, and a third reviewer will be consulted if necessary. Citations returned by the search will be imported into EndNote X7 (Thomas Reuters, Philadelphia, PA, USA).

#### Data extraction

Two reviewers (JPL, DN) will independently extract data from all included citations using a pre-designed electronic form that will be pilot tested using a random sample of 10 citations. Once data are consistently abstracted (κ ≥0.8), [4] reviewers will proceed with full data extraction. We will document the type of citation (e.g. original research), country, study design, study participants (e.g. disease and/or syndrome under investigation), focus of citation (e.g. identify low-value practices) recruitment and sampling and reference standard. For each barrier and facilitator we will document the name (e.g. lack of knowledge), type of practice (e.g. therapeutic vs. diagnostic, drug vs. non-drug), setting (specialty of unit), documented clinical application, the primary outcome (e.g. mortality, length of stay, days free of a particular organ failure), proposed interventions and the magnitude of the intervention’s effect (actual effect measure related to the primary outcome), and subsequent conclusions drawn by the authors. Any eligibility disagreements encountered during data extraction will be resolved by consensus, or arbitration by a third reviewer. Agreement between reviewers will be quantified using the κ statistic [4].

#### Quality assessment

For original research studies, the same two reviewers will independently assess the quality of their methodology assessed using the framework of Caldwell et al., [31] for evaluating both quantitative and qualitative study designs.

#### Analysis

We will present a narrative synthesis of the results. Quantitative and qualitative analyses will be performed. Agreement on data abstraction and article classification will be assessed with Cohen κ reliability coefficients [32]. Quantitative analysis will include summaries of the articles using counts, proportions, mean (standard deviation), or median (inter-quartile range, IQR) where appropriate. Qualitative analysis will include development of a comprehensive list of barriers and facilitators and proposed interventions identified in the literature and categorized and quantified using simple numerical counts. Translation of key concepts from all studies will be performed to identify novel concepts not explored by individual studies and their overlap, synthesized and refined to identify core themes.

#### Statistical analyses

All statistical analyses will be performed using Stata SE 13.1 (Stata Corp. LP, College Station, TX, USA). Qualitative analyses will be performed using NVivo-10 (QSR International Pty Ltd, Burlington, MA, USA).

#### Outcome

A comprehensive summary of the de-adoption evidence base, including the identification of published barriers, facilitators and associated interventions.

### Stakeholder focus groups (Phase two)

#### Objective

To describe perceptions pertaining to de-adoption among adult acute care stakeholders, including front-line providers (inclusive), managers and decision makers responsible for patient care, and patients and family members in an effort to identify important contextual factors and themes not published in the literature. Patients and family members have been added to this phase of the work because we are particularly interested in their perceptions of low value care, the role they feel they can play in de-adopting low value care practices (if any), and their perceptions of risks and benefits related to de-adoption.

#### Design

Focus groups will be conducted with key stakeholders (e.g. front-line providers (inclusive), managers and decision makers responsible for patient care and patient and family members) in four Strategic Clinical Networks (Emergency, Cardiovascular Health and Stroke, Surgery and Critical Care) to elicit their perspectives of barriers and facilitators to de-adoption from the four areas of acute care medicine we are targeting. The focus group methodology was selected for this phase of work because it is an effective technique for exploring impressions and experiences and the contextual factors that influence those perspectives [33]. Focus groups use interaction to build upon individual comments while encouraging the expression of unique thoughts, creating a synergy of ideas not available in one-on-one interviews [33].

#### Setting

We will conduct 3 focus groups (divided by providers, decision makers, and patients/family members) with adult acute care stakeholders in each of the four Strategic Clinical Networks (*n* = 12). Each network will nominate participants. Barriers, facilitators and associated interventions identified in *Phase one* will broadly be used to inform focus group guides. Moreover, an example for de-adoption of two practices—one related to harm and one related to no benefit—will be included to help frame discussion. This is important because the barriers and facilitators may be different between low-value and harmful practices. Customized practice examples will be chosen in each area of acute care medicine.

#### Sampling & recruitment

Focus groups rely on purposive samples, chosen in accordance with the project’s goals [34]. We anticipate that participants may have different experiences relative to the following categories: a) geographic location (urban vs. regional); b) type of institution (teaching vs. non-teaching); and c) area of specialization (Emergency Medicine vs. Cardiovascular Health and Stroke vs. Surgery vs. Critical Care); d) stakeholder group (provider vs. decision maker vs. patient and family member). If variability related to resources, processes of care and institutional policies contribute to different impressions of de-adoption; these categories have a high likelihood of bringing them to light. Because different backgrounds are expected to correspond to different perspectives on de-adoption, we propose conducting specialty-specific focus groups. This should ensure that we capture a diversity of views, while maintaining the richness of perspective related to each area of specialization. We will not, however, separate focus groups by type of institution or geographic location, as we anticipate that any comparisons that might flow from these within-specialty differences will help to generate a richer more in-depth discussion. Thus, a total of 12 focus groups involving 96–120 participants (8–10/group) are planned. Participants will be engaged according to standard principles of qualitative research [35]. A convenience sample of consecutive consenting acute care stakeholders, including patient and family members, front-line providers and managers and decision makers responsible for patient care from each of the four areas of specialization will be recruited.

Stakeholders will be eligible if they are currently affiliated with an acute care setting (or in the case of patients and family members, have had previous experiences in acute care) and if their spoken language is English. Written informed consent will be obtained prior to commencement of the focus group. Focus groups will be moderated by a researcher trained in qualitative methods (JPL), and a semi-structured focus group guide designed to elicit participants’ perceptions of barriers and facilitators to de-adoption in the acute care setting they are most affiliated with will be followed. Each focus group will include an overview to introduce the purpose and agenda, an icebreaker exercise to familiarize the participants with each other, a series of questions which proceed from general to specific, and a summary to highlight and verify key points [33]. Domains of inquiry will be identified from the systematic review and discussion among the investigators. The focus group guide will be pilot-tested on a small group of specialty-specific stakeholders to refine the wording and flow of questions. Because we will use an iterative approach to data analysis, questions will likely be added or subtracted as the study progresses.

#### Analysis

Focus groups will be audio taped, transcribed verbatim, de-identified, imported into NVivo-10 for data management and independently coded by two investigators with qualitative research experience, drawing on qualitative content analysis to identify themes and sub-themes. All focus group participants will be provided with a copy of the study report to review and comment on as a form of member-checking. There are no sample size considerations per se for the qualitative component of the proposed study. We will develop graphical summaries of the data to identify when asymptotes in data collection have been reached and no new themes (i.e. barriers and facilitators) are identified, indicating that the boundaries of the phenomenon have been tapped.

#### Outcome

A description of stakeholder perceptions of de-adoption and the identification of important themes not published in the literature.

### Stakeholder surveys (Phase three)

#### Objective

To determine which of the barriers and facilitators identified through the systematic review and focus groups are most relevant to frontline providers working in adult acute care settings.

#### Design

Individual stakeholder surveys will be conducted with a representative sample of front line providers (e.g. nurses (bedside, clinician and practitioner), physicians (attending and resident), pharmacists), and those in leadership positions (e.g., medical director, unit manager) across 14 adult acute care facilities in Alberta. Survey items will be generated from the barriers and facilitators identified in the systematic review and focus groups, extending the generalizability of those findings. Using a 7-point Likert scale, survey respondents will be asked to rate barriers and facilitators according to their importance as opportunities for improving the de-adoption of low-value clinical practices (ineffective, harmful) within their specific settings (Emergency Department, Operating Room, Cardiovascular Coronary Care Unit and Intensive Care Unit) and suggest additional ideas using open response questions. Using a similar scale, providers will also be asked to rate proposed interventions according to their perceived level of effectiveness to initiate and sustain practice change within their specific clinical setting. All respondents will be asked to identify the top 5 barriers and facilitators that are most important in their clinical setting for the de-adoption of ineffective practices, the top 5 for harmful practices, and the top 5 interventions. We will request identifiable data in the survey (age, gender, number of years working in acute care, primary specialization, role in organization, type of institution currently working in) to determine whether and how perceptions differ by practice setting, hospital type (tertiary vs. community vs. regional) and provider characteristics (e.g. MDs vs. RNs vs. R.PH).

#### Sampling & recruitment

A stratified random sample (using the characteristics outlined below) of frontline providers across the provincial acute care spectrum (*n* = 2,285 providers sampled) will be targeted using a clear and easy to understand electronic survey (SurveyMonkey, Palo Alto, CA, USA). Our sampling frame will be developed from the frontline membership lists of the participating units (Emergency, Cardiovascular Health and Stroke, Surgery, Critical Care) and electronic surveys will be sent to potential respondents via email. Our targets include 400 completed surveys from each of the four target areas of acute care medicine (*n* = 1,600), with relatively equal distribution across hospital (*n* = 14), provider and unit characteristics (~30 surveys per unit from each of the 14 hospitals).

Our goal for survey recruitment is to capture a diverse representation of frontline providers, including nurses (bedside, clinician and practitioner), physicians (attending and resident), pharmacists and those in leadership positions (unit manager, member of the executive) with different characteristics (tertiary & community, teaching & non-teaching, variation in specialization) to generate a representative list of barriers and facilitators to de-adoption. Three survey reminders (two weeks apart) will be sent to everyone in the sampling frame.

#### Analysis

Quantitative analyses will describe barriers (e.g. culture resistant to change) and facilitators (e.g. local champion) to de-adoption reported in the individual surveys, resulting in a comprehensive list. We will present global data to describe the importance of each barrier and facilitator using medians and interquartile range. We will also report the proportion of knowledge users that indicated specific barriers and facilitators as being among the top 5 most important along with their respective binomial 95% confidence. We will present detailed tabulations of survey responses by provider (RN, MD, R.PH), hospital (tertiary vs. community, teaching vs. non-teaching) and practice setting (e.g. surgery vs. critical care) characteristics. The significance of observed differences in reported barriers and facilitators will be evaluated using Chi-square tests and the analysis of variance (ANOVA). It is anticipated that we will perform quantitative analyses on approximately 1,600 stakeholder surveys (2, 285 surveys offered with 70% response rate targeted) that will provide binomial confidence intervals of ±2.4%.

#### Outcome

Determination of which facilitators, barriers and interventions identified in the systematic review and focus groups are most relevant to frontline providers currently working in acute care.

### Main deliverable

Data from all three phases of work will be synthesized to inform the development of an *applied framework to guide the process of de-adoption* across the spectrum of acute care medicine*.* Specifically, barriers and facilitators to de-adoption identified through this work will be catalogued and differentiated by their applicability to practice setting (Emergency Department, Operating Room, Cardiovascular Coronary Care Unit and Intensive Care Unit), hospital characteristics (tertiary vs. community vs. regional), provider characteristics (MD vs. RN vs.R.PH) and the reason for de-adoption (lack of efficacy vs. potential to cause harm). We will then integrate findings with a developed list of targeted interventions (e.g. audit and feedback) to guide the process of de-adoption in each of the four areas of specialization (Emergency vs. Cardiovascular Health and Stroke vs. Surgery vs. Critical Care). The draft framework will be circulated to focus group or survey participants who indicate interest, for feedback and as a form of member-checking. While a number of lists outlining low-value practices in acute care medicine currently exist, [36, 37] this applied framework will map barriers and facilitators to de-adoption, allowing stakeholders to effectively utilize these lists to initiate, execute and sustain an evidence-based process.

## Discussion

### Relevance of findings

Low-value clinical practices that are ineffective or potentially harmful to patients are common in healthcare, in part because implementing scientific evidence into clinical practice is often a difficult and complex process [4]. This study is essential because it will determine barriers and facilitators to the de-adoption of low-value clinical practices in acute care medicine, and provide hospitals with an applied framework to guide the de-adoption process. Ensuring that patients receive the right care, at the right time is the ultimate goal of this study. This is especially important for the population of patients who enter into the acute care system requiring urgent life-or-limb saving treatment in a time sensitive manner. This project will help to build a foundation for the creation of effective strategies to improve the use of best-evidence by facilitating the removal of practices that are no longer supported by best evidence. This study is directly aligned with important system and healthcare needs to increase quality of care and value for money by facilitating the removal of unnecessary, ineffective and potentially harmful treatments and technologies in acute care medicine.

### Next steps

This study has the potential to improve the care that acutely ill patients receive during their hospital stay by identifying and cataloguing top barriers and facilitators to the de-adoption of low-value and harmful clinical practices to create an applied framework to guide the de-adoption process in acute care medicine. This is a first step towards improving patient care. We intend to test and implement our de-adoption strategy through a series of future projects: **(1)** evaluate the framework by pilot testing one KT intervention to de-adopt a single harmful or ineffective clinical practice that is relevant to each of the four areas of acute care included in this study (e.g. reducing the use of albumin for fluid resuscitation); **(2)** compare the impact of our de-adoption strategy for practices that are low-value vs. those that are harmful to patients; **(3)** Examine the relationship between de-adoption and long-term clinical and economic outcomes in a prospective cohort study.

## Appendix

### Medline search (October 25, 2016)

1. ((abandon* or contradict* or refute* or refuting or reassess* or re-assess* or obsole* or revers* or delist* or de-list* or disinvest* or dis-invest* or discontinu* or dis-continu* or decommission* or de-commission* or deadopt* or de-adopt* or de-implement* or deimplement* or “health care’ withdraw*” or (no adj benefit*)) adj5 (healthcare or technolog* or device* or intervention* or health practi?e* or medical or medical practi?e* or procedur* or drug or drugs or biotechnology*)).tw.
2. limit 1 to English language
3. limit 2 to animals
4. limit 2 to (animals and humans)
5. 3 not 4
6. 2 not 5
7. limit 6 to (“all infant (birth to 23 months)” or “newborn infant (birth to 1 month)” or “infant (1 to 23 months)” or “preschool child (2 to 5 years)” or “child (6 to 12 years)”)
8. 6 not 7
9. Limit 8 to yr= “2014-Current”

## Additional file

Additional file 1: PROSPERO Registration. (PDF 52 kb)
