# Supplementary material for: Developing a framework to guide the de-adoption of low-value clinical practices in acute care medicine: a study protocol
Source: BMC Health Serv Res. 2017 Jan 19;17:54. doi: 10.1186/s12913-017-2005-x (PMC5247804; doi:10.1186/s12913-017-2005-x)
Supplement: Additional file 1: — PROSPERO Registration. (PDF 52 kb) [file 12913_2017_2005_MOESM1_ESM.pdf]

University of York
Centre for Reviews and Dissemination

NHS
National Institute for Health Research

PROSPERO International prospective register of systematic reviews

Review title and timescale

1 Review title

Give the working title of the review. This must be in English. Ideally it should state succinctly the interventions or exposures being reviewed and the associated health or social problem being addressed in the review.

A systematic review of barriers and facilitators to the de-adoption of low-value clinical practices in adult acute care medicine

2 Original language title

For reviews in languages other than English, this field should be used to enter the title in the language of the review. This will be displayed together with the English language title.

3 Anticipated or actual start date

Give the date when the systematic review commenced, or is expected to commence.

31/10/2016

4 Anticipated completion date

Give the date by which the review is expected to be completed.

31/03/2017

5 Stage of review at time of this submission

Indicate the stage of progress of the review by ticking the relevant boxes. Reviews that have progressed beyond the point of completing data extraction at the time of initial registration are not eligible for inclusion in PROSPERO. This field should be updated when any amendments are made to a published record.

The review has not yet started √

Review stage	Started	Completed
Preliminary searches	No	No
Piloting of the study selection process	No	No
Formal screening of search results against eligibility criteria	No	No
Data extraction	No	No
Risk of bias (quality) assessment	No	No
Data analysis	No	No

Provide any other relevant information about the stage of the review here.

Funded proposal

Review team details

6 Named contact

The named contact acts as the guarantor for the accuracy of the information presented in the register record.

Dr Parsons Leigh

7 Named contact email

Enter the electronic mail address of the named contact.

jeanna.parsonsleigh@albertahealthservices.ca

8 Named contact address

Enter the full postal address for the named contact.

Department of Critical Care Medicine Cumming School of Medicine, University of Calgary Office: 0497-McCaig Tower, Ground Floor 3234 Hospital Drive NW, Calgary, Alberta, T2N 2T9

9 Named contact phone number

Enter the telephone number for the named contact, including international dialing code.

403 944 2488

10 Organisational affiliation of the review

Full title of the organisational affiliations for this review, and website address if available. This field may be completed as 'None' if the review is not affiliated to any organisation.

University of Calgary

Website address:

11 Review team members and their organisational affiliations

Give the title, first name and last name of all members of the team working directly on the review. Give the organisational affiliations of each member of the review team.

Title	First name	Last name	Affiliation
Dr	Jeanna	Parsons Leigh	

12 **Funding sources/sponsors**

Give details of the individuals, organizations, groups or other legal entities who take responsibility for initiating, managing, sponsoring and/or financing the review. Any unique identification numbers assigned to the review by the individuals or bodies listed should be included.

M.S.I Foundation of Alberta

13 **Conflicts of interest**

List any conditions that could lead to actual or perceived undue influence on judgements concerning the main topic investigated in the review.

Are there any actual or potential conflicts of interest?

None known

14 **Collaborators**

Give the name, affiliation and role of any individuals or organisations who are working on the review but who are not listed as review team members.

Title	First name	Last name	Organisation details

Review methods

15 **Review question(s)**

State the question(s) to be addressed / review objectives. Please complete a separate box for each question.

To conduct a systematic review of evidence-based barriers and facilitators to the de-adoption of low-value clinical practices.

16 **Searches**

Give details of the sources to be searched, and any restrictions (e.g. language or publication period). The full search strategy is not required, but may be supplied as a link or attachment.

The search strategy was designed using our existing published scoping review of de-adoption, updating the systematic search and restricting our focus to citations describing barriers and facilitators. To update this review we will search the following electronic databases from March 5, 2014 to Fall 2016: PubMed, MEDLINE, EMBASE, CINAHL and the Cochrane Library. A search of the ‘grey’ literature will also be conducted using the CADTH tool for searching grey literature. Bibliographies of retrieved articles will be searched for additional relevant articles. Additional information will be requested from study authors if necessary. The search terms are specific to articles reporting de-adoption, including text words that include combinations and synonyms of de-adoption and healthcare technologies. Appropriate wildcards will be used to account for plurals and variations in spelling.

17 **URL to search strategy**

If you have one, give the link to your search strategy here. Alternatively you can e-mail this to PROSPERO and we will store and link to it.

18 **Condition or domain being studied**

Give a short description of the disease, condition or healthcare domain being studied. This could include health and wellbeing outcomes.

Any healthcare setting

19 **Participants/population**

Give summary criteria for the participants or populations being studied by the review. The preferred format includes details of both inclusion and exclusion criteria.

We will include English language citations that refer to barriers or facilitators to the de-adoption of any clinical practice in adult patients (age >= 18 years) with medical, surgical, or psychiatric illnesses in any healthcare setting

20 **Intervention(s), exposure(s)**

Give full and clear descriptions of the nature of the interventions or the exposures to be reviewed

Prior to the screening of titles and abstracts, the citation screening form will be calibrated through pilot testing. Two investigators will independently review a random sample of 50 citations from the literature search, and the inclusion/exclusion criteria will be serially revised until citation selection is reliable (k >= 0.8). The same two investigators will then independently review citations through a two-step process. First, the titles and abstracts of all citations will be screened against inclusion criteria. Second, the full text of any citation categorized as include or unclear by either investigator will be reviewed to determine whether it meets eligibility criteria. Eligibility disagreements will be resolved between the reviewers by consensus, and a third reviewer will be consulted if necessary. Citations returned by the search will be imported into EndNote X7 (Thomas Reuters, Philadelphia, PA, USA).

21 **Comparator(s)/control**

Where relevant, give details of the alternatives against which the main subject/topic of the review will be compared (e.g. another intervention or a non-exposed control group).

No control group necessary.

22 **Types of study to be included**

Give details of the study designs to be included in the review. If there are no restrictions on the types of study design eligible for inclusion, this should be stated.

All original and non-original quantitative and qualitative research citations will be eligible.

23 Context

Give summary details of the setting and other relevant characteristics which help define the inclusion or exclusion criteria.

Studies in any healthcare setting.

24 Primary outcome(s)

Give the most important outcomes.

Outcomes are likely to include: barriers, facilitators and interventions designed to target barriers or complement facilitators.

Give information on timing and effect measures, as appropriate.

We define barriers and facilitators as factors that influence the discontinuation of a clinical practice after it has been previously adopted

25 Secondary outcomes

List any additional outcomes that will be addressed. If there are no secondary outcomes enter None.

None

Give information on timing and effect measures, as appropriate.

The search strategy was designed using our existing published scoping review of de-adoption, updating the systematic search and restricting our focus to citations describing barriers and facilitators. To update this review we will search the literature from March 5, 2014 to Fall 2016.

26 Data extraction (selection and coding)

Give the procedure for selecting studies for the review and extracting data, including the number of researchers involved and how discrepancies will be resolved. List the data to be extracted.

Two reviewers will independently extract data from all included citations using a pre-designed electronic form that will be pilot tested using a random sample of 10 citations. Once data are consistently abstracted (Kappa >=0.8), reviewers will proceed with full data extraction. We will document the type of citation (e.g. original research), country, study design, study participants (e.g. disease and/or syndrome under investigation), focus of citation (e.g. identify low-value practices) recruitment and sampling and reference standard. For each barrier and facilitator we will document the name (e.g. lack of knowledge), type of practice (e.g. therapeutic vs. diagnostic, drug vs. non-drug), setting (specialty of unit), documented clinical application, the primary outcome (e.g. mortality, length of stay, days free of a particular organ failure), proposed interventions and the magnitude of the intervention's effect (actual effect measure related to the primary outcome), and subsequent conclusions drawn by the authors.

27 Risk of bias (quality) assessment

State whether and how risk of bias will be assessed, how the quality of individual studies will be assessed, and whether and how this will influence the planned synthesis.

For original research studies, the same two reviewers will independently assess the quality of their methodology assessed using the framework of Caldwell et al., for evaluating both quantitative and qualitative study designs. Any eligibility disagreements encountered during data extraction will be resolved by consensus, or arbitration by a third reviewer. Agreement between reviewers will be quantified using the Kappa statistic.

28 Strategy for data synthesis

Give the planned general approach to be used, for example whether the data to be used will be aggregate or at the level of individual participants, and whether a quantitative or narrative (descriptive) synthesis is planned. Where appropriate a brief outline of analytic approach should be given.

We will present a narrative synthesis of the results. Quantitative and qualitative analyses will be performed. Agreement on data abstraction and article classification will be assessed with Cohen's Kappa reliability coefficients. Quantitative analysis will include summaries of the articles using counts, proportions, mean (standard deviation), or median (inter-quartile range, IQR) where appropriate. Qualitative analysis will include development of a comprehensive list of barriers and facilitators and proposed interventions identified in the literature and categorized and quantified using simple numerical counts. Translation of key concepts from all studies will be performed to identify novel concepts not explored by individual studies and their overlap, synthesized and refined to identify core themes. All statistical analyses will be performed using Stata SE 13.1 (Stata Corp. LP, College Station, TX, USA). Qualitative analyses will be performed using NVivo-10 (QSR International Pty Ltd, Burlington, MA, USA).

29 Analysis of subgroups or subsets

Give any planned exploration of subgroups or subsets within the review. ‘None planned’ is a valid response if no subgroup analyses are planned.

None planned

Review general information

30 Type and method of review

Select the type of review and the review method from the drop down list.

Systematic review

31 Language

Select the language(s) in which the review is being written and will be made available, from the drop down list. Use the control key to select more than one language.

English

Will a summary/abstract be made available in English?

Yes

32 **Country**

Select the country in which the review is being carried out from the drop down list. For multi-national collaborations select all the countries involved. Use the control key to select more than one country.

Canada

33 **Other registration details**

Give the name of any organisation where the systematic review title or protocol is registered together with any unique identification number assigned. If extracted data will be stored and made available through a repository such as the Systematic Review Data Repository (SRDR), details and a link should be included here.

34 **Reference and/or URL for published protocol**

Give the citation for the published protocol, if there is one.

Give the link to the published protocol, if there is one. This may be to an external site or to a protocol deposited with CRD in pdf format.

35 **Dissemination plans**

Give brief details of plans for communicating essential messages from the review to the appropriate audiences.

In addition to producing a report for the funders of this review, a paper will be submitted to a leading journal in this field.

Do you intend to publish the review on completion?

Yes

36 **Keywords**

Give words or phrases that best describe the review. (One word per box, create a new box for each term)

De-Adoption

Low-Value Practices

Barriers

Facilitators

Acute Care Medicine

37 **Details of any existing review of the same topic by the same authors**

Give details of earlier versions of the systematic review if an update of an existing review is being registered, including full bibliographic reference if possible.

This is an update of our team's previously completed scoping review: Niven DJ, Mrklas KJ, Holodinsky JK, et al. Towards understanding the de-adoption of low-value clinical practices: A scoping review. BMC Med 2015;13:255.

38 **Current review status**

Review status should be updated when the review is completed and when it is published.

Ongoing

39 **Any additional information**

Provide any further information the review team consider relevant to the registration of the review.

40 **Details of final report/publication(s)**

This field should be left empty until details of the completed review are available.

Give the full citation for the final report or publication of the systematic review.

Give the URL where available.
